# FGF21 overexpression alleviates VSMC senescence in diabetic mice by modulating the SYK-NLRP3 inflammasome-PPARγ-catalase pathway: FGF21 alleviates high glucose-induced VSMC senescence

**DOI:** 10.3724/abbs.2024032

**Published:** 2024-05-10

**Authors:** Yanyan Heng, Wei Wei, Linzhong Cheng, Feifei Wu, Haoyu Dong, Jingxia Li, Jianing Fu, Bingjie Yang, Xinyue Liang, Chunyan Liu, Haiju Li, Haihua Liu, Pengfei Zhang

**Affiliations:** 1 Department of Nephrology Heping Hospital Affiliated to Changzhi Medical College Changzhi 046000 China; 2 Department of Pharmacology Changzhi Medical College Changzhi 046000 China; 3 Department of Endocrinology Heping Hospital Affiliated to Changzhi Medical College Changzhi 046000 China; 4 Department of Clinical Central Laboratory Heping Hospital Affiliated to Changzhi Medical College Changzhi 046000 China; 5 Department of National Institute for Clinical Trials of Drugs and Phase I Clinical Trial Laboratory Heping Hospital Affiliated to Changzhi Medical College Changzhi 046000 China; 6 Department of Anesthesia Changzhi Medical College Changzhi 046000 China; 7 Department of Stomatology Changzhi Medical College Changzhi 046000 China; 8 Department of Medical Imageology Changzhi Medical College Changzhi 046000 China

**Keywords:** vascular smooth muscle cell, senescence, NLRP3 inflammasome, PPARγ, catalase

## Abstract

Diabetes accelerates vascular senescence, which is the basis for atherosclerosis and stiffness. The activation of the NOD-like receptor family pyrin domain containing 3 (NLRP3) inflammasome and oxidative stress are closely associated with progressive senescence in vascular smooth muscle cells (VSMCs). The vascular protective effect of FGF21 has gradually gained increasing attention, but its role in diabetes-induced vascular senescence needs further investigation. In this study, diabetic mice and primary VSMCs are transfected with an FGF21 activation plasmid and treated with a peroxisome proliferator-activated receptor γ (PPARγ) agonist (rosiglitazone), an NLRP3 inhibitor (MCC950), and a spleen tyrosine kinase (SYK)-specific inhibitor, R406, to detect senescence-associated markers. We find that FGF21 overexpression significantly restores the level of catalase (CAT), vascular relaxation, inhibits the intensity of ROSgreen fluorescence and p21 immunofluorescence, and reduces the area of SA-β-gal staining and collagen deposition in the aortas of diabetic mice. FGF21 overexpression restores CAT, inhibits the expression of p21, and limits the area of SA-β-gal staining in VSMCs under high glucose conditions. Mechanistically, FGF21 inhibits SYK phosphorylation, the production of the NLRP3 dimer, the expression of NLRP3, and the colocalization of NLRP3 with PYCARD (ASC), as well as NLRP3 with caspase-1, to reverse the cleavage of PPARγ, preserve CAT levels, suppress ROSgreen density, and reduce the expression of p21 in VSMCs under high glucose conditions. Our results suggest that FGF21 alleviates vascular senescence by regulating the SYK-NLRP3 inflammasome-PPARγ-catalase pathway in diabetic mice.

## Introduction

Diabetic cardiovascular, cerebrovascular, and peripheral vascular diseases are the main causes of death and disability in diabetic patients [ 1, 2]. The senescence of vascular smooth muscle cells (VSMCs) is the underlying pathological change of vascular calcification, remodeling and stiffening of the vascular wall, and impaired relaxation ability, leading to serious consequences such as myocardial infarction and stroke [ 3– 5]. High glucose (HG) conditions induce senescence in VSMCs, characterized by a phenotypic switch from a contractile phenotype to a secretory phenotype, increased proliferation and migration, excessive collagen secretion, disruption of the microenvironmental balance of the vascular wall, and cytokine-mediated elastin damage, ultimately resulting in vascular sclerosis and impaired relaxation function [ 2, 3, 6– 8]. Therefore, combating hyperglycemia-induced vascular smooth muscle layer senescence is one of the crucial strategies for preventing and treating diabetic vascular diseases and their severe adverse prognosis. Hyperglycemia-induced oxidative stress is a vital process in VSMC senescence. Studies have proven that HG induces the upregulation of reactive oxygen species (ROS) levels, which in turn triggers proliferation and migration, phenotypic switching, and calcification in VSMCs [ 9– 11]. However, there is a relative lack of research on high glucose-induced ROS upregulation during VSMC senescence.

Recent studies have shown that activation of the NOD-like receptor family pyrin domain-containing 3 (NLRP3) inflammasome plays a key role in ROS-mediated VSMC senescence [ 12, 13]. However, further research is still needed to understand the relationship between the NLRP3 inflammasome and ROS production. The NLRP3 inflammasome is an important component of the innate immune system and has been shown to be associated with numerous major human diseases [14]. Studies have shown that NLRP3 inflammasome activation leads to dysfunction of endothelial cells (ECs) and VSMCs, as well as DNA damage-mediated senescence in these cells [ 12, 15]. Moreover, our previous study suggested that NLRP3 inflammasome activation is likely one of the key mechanisms inducing vascular senescence in the diabetic environment [16]. However, the mechanisms by which the HG environment activates the NLRP3 inflammasome remain uncertain. The present study revealed that the generation of NLRP3 dimers in response to HG represents a potential early event in inflammasome activation.

Fibroblast growth factor 21 (FGF21) is predominantly expressed in the liver and acts systemically through secretion into the bloodstream as a cytokine; it plays a role in regulating glucose and lipid metabolism, improving insulin sensitivity, suppressing appetite, and reducing the preference for sweet foods [ 17, 18]. FGF21 is considered a potential novel therapy for type 2 diabetes and nonalcoholic fatty liver disease [17]. Our previous studies showed that FGF21 downregulates NLRP3 inflammasome activity, inhibits VSMC proliferation and migration, and alleviates diabetes-aggravated neointimal hyperplasia [19]. Some studies have demonstrated that FGF21 alleviates senescence of human brain vascular smooth muscle cells by regulating the adenosine monophosphate-activated protein kinase (AMPK)-p53 pathway [20]. However, it remains uncertain whether FGF21 has similar alleviating effects on vascular senescence induced by the diabetic environment, particularly in terms of its protective effects on vascular smooth muscle layer senescence, which is still extremely lacking.

In this study, we describe how FGF21 reduces vascular smooth muscle layer senescence by inhibiting NLRP3 inflammasome-dependent oxidative stress in diabetic mice.

## Materials and Methods

### Ethics approval and consent to participate

Animal handling and experimental procedures were approved by the Ethics Committee of Changzhi Medical College (DW2022053) following the guidelines of the US National Institutes of Health and the Animal Research Reporting *In Vivo* Experiments (ARRIVE).

### Primary VSMC isolation and culture

According to our previous studies [19], primary VSMCs were isolated from wild-type (WT) mice (20–22 g, 9 weeks; purchased from GemPharmatech, Nanjing, China) using the tissue block adhesion method. Briefly, the mouse was sacrificed, the aorta was quickly removed without tearing, the adventitia was gently peeled off under an MSD540T operating microscope (Murzider, Dongguan, China), the remaining vessel was opened longitudinally, the endothelium was scraped out, and the remaining piece was cut into tissue blocks approximately 3 mm square. The tissue blocks were seeded with media and cultured in Dulbecco’s modified Eagle’s medium (DMEM; Gibco, Shanghai, China) supplemented with 15% fetal bovine serum (FBS; Gibco), 100 IU/mL penicillin and 100 μg/mL streptomycin (C0222; Beyotime, Shanghai, China) at 37°C in a 5% CO _2_ humidified incubator. After reaching confluence, the VSMCs were passaged and cultured in regular glucose DMEM (5 mM) or high-glucose DMEM (30 mM, HG; Gibco) containing 10% FBS, 100 IU/mL penicillin and 100 μg/mL streptomycin at 37°C in the 5% CO _2_ humidified incubator. Before measurement of the senescence indicators, VSMCs were induced with HG for more than 72 h as described in a previous study [16].

### Transfection

Primary VSMCs from diabetic mice (db/db, ~30 g, 9 weeks; purchased from GemPharmatech) and their littermates were transfected with control plasmid (null-ACT, sc-437275; Santa Cruz, Shanghai, China) or FGF21 activated plasmid (sc-425248-ACT; Santa Cruz). The plasmids were diluted in transfection reagent (18668-11-2, Entranster ^TM^-in vivo; Engreen, Beijing, China) and intravenously injected into mice at a dosage of 5 μg/week [ 21, 22]. Aortas were harvested on day 90.

Transfection of VSMCs was performed using electroporation. Plasmids were diluted in electroporation-specific reagent (98668-20, Entranster ^TM^-E; Engreen). VSMCs (10 ^5^) were treated with 4 μg of plasmid and subjected to one electric shock at 150 V using a gene introduction instrument (SCIENTZ-2C; Scientz, Ningbo, China). After being left to stand for 2 min, the cells were inoculated into the culture medium.

### Immunofluorescence analysis

The transfected diabetic mice and their littermates were maintained for 90 days and then sacrificed. Rosiglitazone (RSG; 5mg/kg, HY-17386; MCE) and R406 (5mg/kg, HY-12067; MCE) were orally administered, and MCC950 (10 mg/kg, HY-12815A; MCE) was administered by intraperitoneal injection. RSG is a known PPARγ agonist [23], MCC950 is a potent, selective NLRP3 inhibitor [24], and R406 is a specific spleen tyrosine kinase (SYK) inhibitor [25]. The aortas were collected, and the adventitia was removed. Aortas were fixed with 4% paraformaldehyde (PFA) and then cut at 5 μm with a pathological microtome (KD2260; KEDI, Hangzhou, China). The sections were blocked with 5% BSA after antigen retrieval using improved citrate antigen retrieval solution (P0083; Beyotime). The sections were incubated with primary antibodies including rabbit anti-catalase (1:100, CY6783; Abways, Shanghai, China), Waf1/Cip1/CDKN1A p21 antibody (sc-6246; Santa Cruz), mouse anti-NLRP3 (1:100, 68102-1-Ig; Proteintech, Wuhan, China), rabbit anti-TMS1 (ASC; 1:100, CY5689; Abways), and rabbit anti-caspase-1 (p20; 1:50, WL02996a; Wanleibio, Shenyang, China) overnight at 4°C, and then with goat anti-mouse IgG (H+L) Alexa Fluor 594 (1:100, AB0152; Abways) or goat anti-rabbit IgG (H+L) Alexa Fluor 488 (1:100, AB0141; Abways) secondary antibody. The nuclei were stained with 4′,6-diamidino-2-phenylindole (DAPI; C1006; Beyotime). Images were obtained with an FRD-6C fluorescence microscope (Cossim, Beijing, China). The integrated density of the immunofluorescence signal was calculated by Image J software (National Institutes of Health, Bethesda, USA).

VSMCs (5×10 ^4^ cells) were seeded in 35-mm dishes. After treatment with regular glucose, HG, RSG (10 μM), MCC950 (15 nM), or R406 (2.5 μM), the cells were washed three times with PBS containing 137 mM NaCl, 2.7 mM KCl, 10 mM Na _2_HPO _4_, 1.8 mM KH _2_PO _4_ and fixed with precooled ethanol for 30 min. The cell membrane was permeablized with 0.3% Triton X-100 (BS084; Biosharp, Hefei, China) for 15 min, and the cells were blocked with 5% bovine serum albumin (BSA; A8010; Solarbio, Beijing, China) for 1.5 h. Then, the cells were incubated with primary antibodies, including mouse anti-NLRP3 (1:100), rabbit anti-PYCARD (ASC, 1:100) and rabbit anti-caspase-1 (p20, 1:50) overnight at 4°C, and then with goat anti-mouse IgG (H+L) Alexa Fluor 594 (1:200) or goat anti-rabbit IgG (H+L) Alexa Fluor 488 (1:100) secondary antibody for 2 h at room temperature. DAPI was used to label the nuclei. Fluorescent images were photographed and processed as above.

### Hydrogen peroxide detection

The accumulation of hydrogen peroxide in aortas and VSMCs was detected by staining with ROSgreen (MX5202; Maokangbio, Shanghai, China), a specific hydrogen peroxide probe [ 26, 27]. ROSgreen was first dissolved in DMSO and then diluted with HEPES solution (C0215; Beyotime). The mice were intravenously injected with a diluted ROSgreen solution (20 μM) and were sacrificed after 1 h. The aortas were then removed, the adventitia was peeled off, and the ROSgreen fluorescence was detected using the MSD540T operating microscope. VSMCs were treated with a diluted ROSgreen solution (5 μM) and incubated for 20 min. The cells were washed and fixed, and ROSgreen fluorescence was detected. The integrated density of ROSgreen fluorescence was calculated using Image J software.

### Senescence-associated β-galactosidase (SA-β-gal) staining

The accumulation of SA-β-gal was detected using a commercial SA-β-Gal staining kit (C0602; Beyotime). Briefly, aortas without adventitia were removed, and VSMCs were fixed immediately after treatment, washed with PBS three times, and stained with SA-β-gal staining solution. Images of aortas were captured with an MSD540T operating microscope and images of cells were captured with an FRD-6C inverted microscope (Cossim, Beijing, China). The area of SA-β-gal staining (green staining) was calculated using Image J software.

### Masson staining

Collagen accumulation in aortas without adventitia was measured using a Masson staining kit (WLA045; Wanleibio, Shenyang, China). Images of the sections were captured with the FRD-6C inverted microscope. The area of collagen staining (blue staining) was calculated using Image J software.

### Western blot analysis

Western blot analysis was performed as described previously [19]. Total protein, nuclear protein, and mouse vascular protein were extracted from the cells using the corresponding protein extraction kits (P0033, P0027, P0013M; Beyotime), respectively. The protein concentration was quantified by using a BCA Protein Assay Kit (P0010; Beyotime). The primary used were rabbit anti-NLRP3 (1:1000, CY5651; Abways), rabbit anti-pSyk-try525 (1:500, AF8404; Affinity, Changzhou, China), rabbit anti-Syk (1:500, CY3461; Abways), rabbit anti-PPARγ (1:500, WL01800; Wanleibio, Shenyang, China), and rabbit anti-α-tubulin (1:4000, AB0048; Abways). The secondary antibody was HRP-conjugated goat anti-rabbit IgG (1:4000, AB0101; Abways). Densitometric analysis of the blot images was performed with Image J software.

### Vascular tension recording

The relaxation function of the aortas was detected by a tension detection system (BL-420S; TaiMeng, Chengdu, China) as described previously [ 16, 19]. The mice were anesthetized, and the aortas were quickly removed and immersed in Krebs Henseleit (KH) solution (pH 7.4, 119 mM NaCl, 25 mM NaHCO _3_, 11.1 mM glucose, 2.4 mM CaCl _2_, 4.7 mM KCl, 1.2 mM KH _2_PO _4_, 1.2 mM MgSO _4_, 0.024 mM Na _2_EDTA). Aortas were carefully dissected into transparent tubes and then cut into vascular rings of approximately 2 mm in width. The endothelium of the aortas was removed using a flexible wire (0.38 mm in diameter). The vascular rings were then suspended in a water-jacketed tissue bath, and the tension was tested. KH solution was maintained at 37°C, and mixed gas containing 95% O _2_ and 5% CO _2_ was continuously bubbled through the bath. When the tension of the rings stabilized at the basal level, the aortic rings were contracted with phenylephrine (Phe; 1 μM, S161304; Aladdin, Shanghai, China) to obtain a maximal response, and the rings were assessed for relaxation function using sodium nitroprusside (SNP; 1×10 ^‒9^ to 1×10 ^‒5^ M, S305727; Aladdin). The record of relaxation induced by SNP (1×10 ^‒4^ M) in the Ctrl group was set as 100% response to the SNP.

### Immunohistochemistry (IHC) of VSMCs

VSMCs (5×10 ^4^ cells) were seeded in 35-mm dishes. After treatment, the VSMCs were fixed with precooled ethanol, permeablized, and blocked as described in immunofluorescence analysis. Then, the cells were incubated with the primary antibodies, including rabbit anti-catalase (1:200) and Waf1/Cip1/CDKN1A p21 (1:100) overnight at 4°C, followed by incubation with HRP-conjugated goat anti-rabbit IgG (1:200, AB0101; Abways) or goat anti-mouse IgG (1:200, AB0102; Abways) secondary antibodies for 1.5 h. 3,3′-Diaminobenzidine (DAB; PH0728; Phygene, Fuzhou, China) was used as a chromogen.

### Detection of active caspase-1, IL-1β, IL-18, and FGF21 levels

Caspase-1 activity was assessed using a commercially available Caspase-1 Activity Assay Kit (C1101; Beyotime) following previously established protocols [28]. The corresponding ELISA kits were used to detect the levels of IL-1β (SEKM-0002; Solarbio), IL-18 (SEKM-0019; Solarbio), and FGF21 (D721010; Sangon Biotech, Shanghai, China) in serum and aorta (with the endothelium and adventitia removed) homogenates, respectively. Arterial homogenates and VSMC lysates were centrifuged at 16,000 *g* for 15 min at 4°C. The supernatants were collected and quantified with a BCA assay kit (P0010; Beyotime). Caspase-1 activity in an equal amount of protein, approximately 200 μg, was determined immediately. Ac-YVAD-pNA was added to the supernatant and incubated for 60‒120 min at 37°C. When the solution exhibited a distinct yellow color, the absorbance of samples were measured using a microplate reader (Thermo Fisher Scientific, Waltham, USA) at 405 nm. The detection of IL-1β, IL-18, and FGF21 was performed according to the manufacturer’s instructions, and the optical density (OD) was measured at 450 nm.

### Statistical analysis

Statistical analysis was performed with Graphpad PRISM 9.0 software. Data are presented as the mean±SE. Significant differences between and within multiple groups were examined using ANOVA for repeated measures, followed by Duncan’s multiple-range test. Independent-Samples *t*-test was used to detect significant differences between two groups. *P*<0.05 was considered statistically significant.

## Results

### Overexpression of FGF21 inhibits SYK phosphorylation and NLRP3 inflammasome activation in the aortas of diabetic mice and HG-induced VSMCs

**Figure 1:**
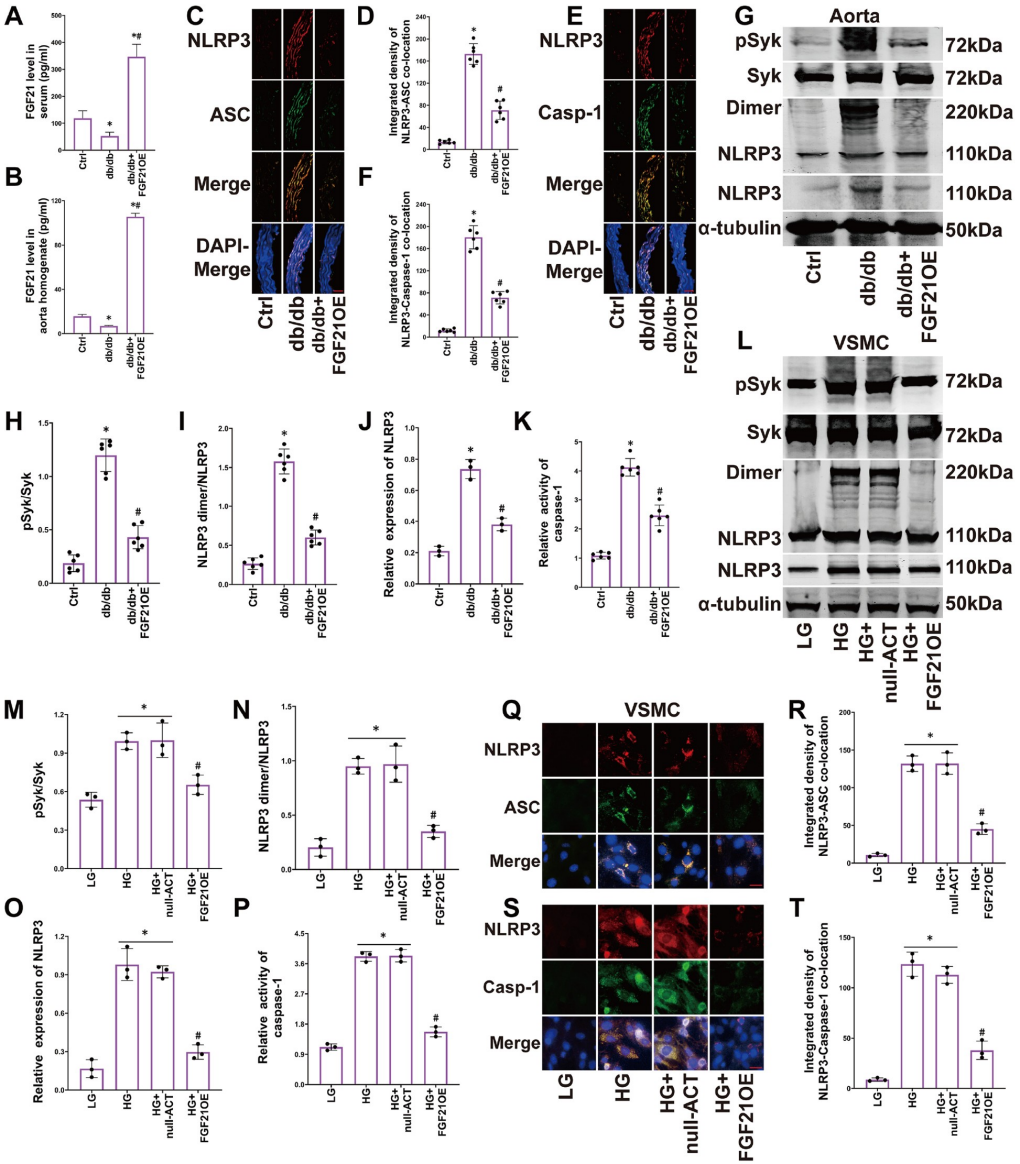
Figure 1 Overexpression of FGF21 inhibits SYK phosphorylation and NLRP3 inflammasome activation in the aortas of diabetic mice and HG-induced VSMCs (A,B) FGF21 levels in serum and aortic tissue homogenates detected by ELISA. (C‒F) Representative immunofluorescence images (400×, scale bar: 20 μm) and summarized integrated density of NLRP3 and ASC, NLRP3 and caspase-1 colocalization in aortas. (G‒J) Representative western blot images and summarized data showing the phosphorylation of SYK, the production of NLRP3 dimers, and the expression of NLRP3 in aortas. (K) The summarized data showing the activity of caspase-1 in aortas. (L‒O) Representative western blot images and summarized data showing the phosphorylation of SYK, the production of the NLRP3 dimer, and the expression of NLRP3 in VSMCs. (P) The summarized data showing the activity of caspase-1 in VSMCs. (Q‒T) Representative immunofluorescence images and summarized integrated density of NLRP3 and ASC, NLRP3 and caspase-1 colocalization (800×, scale bar: 10 μm) in VSMCs. n=6 in mice; n=3 in VSMCs. *P<0.05 vs Ctrl; #P<0.05 vs db/db or HG group. In the animal experiment: Ctrl, wild-type mice from the same litter that were not subjected any intervention; db/db, db/db mice that were not subjected to any intervention; FGF21OE, db/db mice that received intravenous injection of the FGF21-activating plasmid. In the VSMC experiment: LG, cells with regular glucose (5 mM); HG, cells with high glucose (30 mM); HG+null-ACT, cells treated with HG and transfected with the empty vector through electroporation; FGF21OE, cells treated with HG and transfected with the FGF21-activating plasmid through electroporation.

By measuring the levels of FGF21 in both serum and vascular tissue homogenates, we observed that injection of the FGF21 overexpression (FGF21OE) plasmid led to an increase in FGF21 levels in both mouse serum and the vascular smooth muscle layer ( Figure 1A,B). Additionally, we verified that the expression of FGF21 was activated in both mouse aortas and VSMCs ( Supplementary Figure S1A,B,I,J). These results demonstrate that FGF21OE plasmid intervention can increase the levels of FGF21 in the serum and vascular wall of diabetic mice.

To investigate the effect of FGF21 on NLRP3 inflammasome activation induced by diabetes, we assessed the extent of NLRP3 inflammasome activation in the aortas of diabetic mice and smooth muscle cells. In diabetic mice, the colocalization of NLRP3 (red) with ASC (green, also known as PYCARD; TMS1, a bridging adaptor protein of the inflammasome) ( Figure 1C,D) and the colocalization of NLRP3 (red) with caspase-1 (green, also known as IL-1 converting enzyme, a core effector of the NLRP3 inflammasome) ( Figure 1E,F) were found to be increased. Moreover, the phosphorylation of SYK in blood vessels was increased ( Figure 1G,H), the NLRP3 dimer was produced ( Figure 1G,I), the levels of NLRP3 were increased ( Figure 1G,J), the activity of caspase-1 was increased ( Figure 1K), and active IL-1β and IL-18 were also produced ( Supplementary Figure S1C–H). Overexpression of FGF21 inhibited the colocalization of NLRP3 with ASC the colocalization of NLRP3 with caspase-1, the phosphorylation of SYK, NLRP3 dimerization, NLRP3 expression, caspase-1 activity and active IL-1β and IL-18 levels in diabetic aortas ( Figure 1C–K and Supplementary Figure S1C–H).

The smooth muscle layer is the main structural constituent of blood vessels, so we detected the activation of the NLRP3 inflammasome in VSMCs under HG conditions. Similar to the results observed in blood vessels, the HG environment caused the upregulation of SYK phosphorylation in VSMCs ( Figure 1L,M), as well as NLRP3 dimerization ( Figure 1L,N). NLRP3 expression was also upregulated ( Figure 1L,O), along with an increase in the activity of caspase-1 ( Figure 1P) and the production and secretion of active IL-1β and IL-18 ( Supplementary Figure S1K–P). Additionally, HG induced the colocalization of NLRP3 with ASC ( Figure 1Q,R), as well as the colocalization of NLRP3 with caspase-1 ( Figure 1S,T). Treatment with the FGF21 plasmid partially reversed these changes ( Figure 1L–T and Supplementary Figure S1K–P).

These findings suggest that FGF21 prevents hyperglycemia-induced NLRP3 inflammasome assembly and activation in the smooth muscle layer of diabetic mouse aortae and that the inhibition of SYK phosphorylation and NLRP3 dimerization may play a key role in this process.

### Overexpression of FGF21 alleviates senescence in diabetic aortas and HG-treated VSMCs

**Figure 2:**
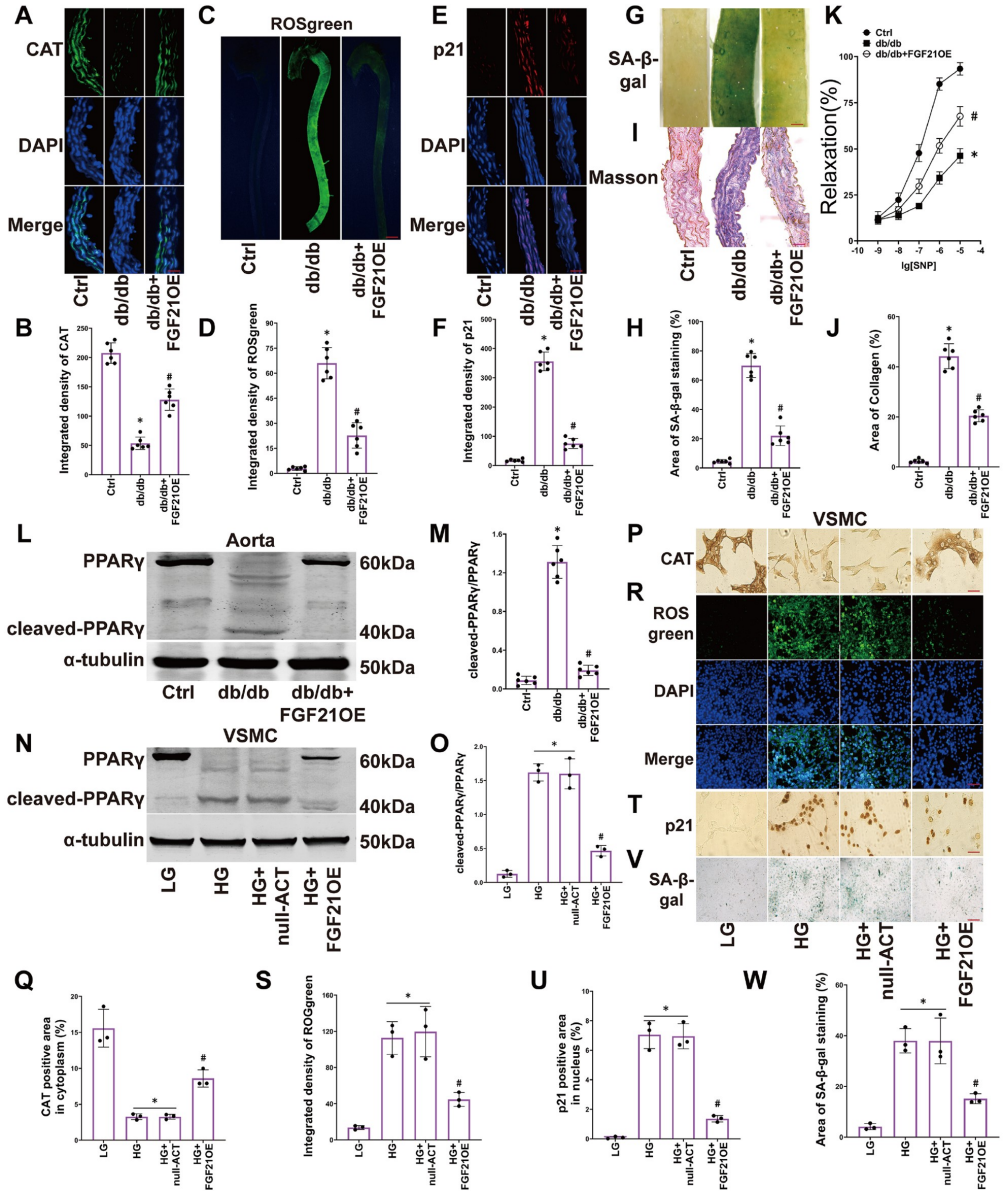
Figure 2 Overexpression of FGF21 alleviates the senescence of the smooth muscle layer in diabetic aortas and HG-treated VSMCs (A‒J) Representative images and summarized data of CAT immunofluorescence (400×, scale bar: 20 μm), ROSgreen staining (15×, scale bar: 1000 μm), p21 immunofluorescence (400×, scale bar: 20 μm), SA-β-gal staining (40×, scale bar: 200 μm) (percentage of green area), and Masson staining (400×, scale bar: 20 μm) (percentage of blue area) in aortas. (K) The summarized data of vascular response to sodium nitroprusside (SNP; 1×10–9 to 1×10–5 M, vascular relaxation) were determined in aorta rings. (L‒M) Representative western blot images and the summarized data show the cleavage of PPARγ in aortas. (N‒O) Representative western blot images and the summarized data show the cleavage of PPARγ in VSMCs. (P‒W) Representative IHC images (400×, scale bar: 20 μm) of CAT levels and summarized data (percentage of yellow area), fluorescence images of ROSgreen staining (200×, scale bar: 40 μm) and summarized data, IHC images (400×, scale bar: 20 μm) of p21 levels and summarized data (percentage of yellow area), SA-β-gal staining. n=6 in mice; n=3 in VSMCs. *P<0.05 vs Ctrl; #P<0.05 vs db/db or HG group.

We measured the expression of catalase (CAT), the accumulation of hydrogen peroxide (ROSgreen, a specific probe for detecting hydrogen peroxide accumulation), the expression of the cellular senescence marker p21, SA-β-gal staining, collagen accumulation, and relaxation ability in the aortas. We found that the CAT level decreased in the aortas of diabetic mice ( Figure 2A,B), the fluorescence intensity of ROSgreen increased ( Figure 2C,D), the p21 expression level increased ( Figure 2E,F), the area of SA-β-gal staining (green staining) increased ( Figure 2G,H), the area of blue-colored collagen accumulation increased ( Figure 2I,J), and the relaxation ability of diabetic aortas decreased ( Figure 2K). Additionally, PPARγ underwent cleavage, resulting in the formation of a 40 kDa fragment ( Figure 2L,M). Overexpression of FGF21 restored CAT levels, inhibited ROSgreen fluorescence, reduced p21 expression, decreased SA-β-gal staining and collagen deposition areas, preserved relaxation ability, and limited cleaved-PPARγ production in the aortas of diabetic mice ( Figure 2A–M).

Furthermore, we examined the level of senescence in primary VSMCs under HG conditions. We found that HG caused the cleavage of PPARγ in VSMCs ( Figure 2N–O). HG impaired the expression of CAT ( Figure 2P,Q) while increasing the fluorescence intensity of ROSgreen ( Figure 2R,S) and the expression of p21 ( Figure 2T,U). HG also increased the area of SA-β-gal staining ( Figure 2V,W) in VSMCs. Overexpression of FGF21 inhibited PPARγ cleavage and partially reversed the changes in VSMC senescence ( Figure 2N–W).

These results demonstrate that the overexpression of FGF21 protects CAT levels, mitigates vascular hydrogen peroxide accumulation, alleviates senescence in VSMCs, and protects the relaxation ability of blood vessels. The protective effects of FGF21 may be associated with its protective effect on PPARγ.

### FGF21 activates the PPARγ-CAT pathway to alleviate senescence in the vascular smooth muscle layer of diabetic mice and HG-treated VSMCs

**Figure 3:**
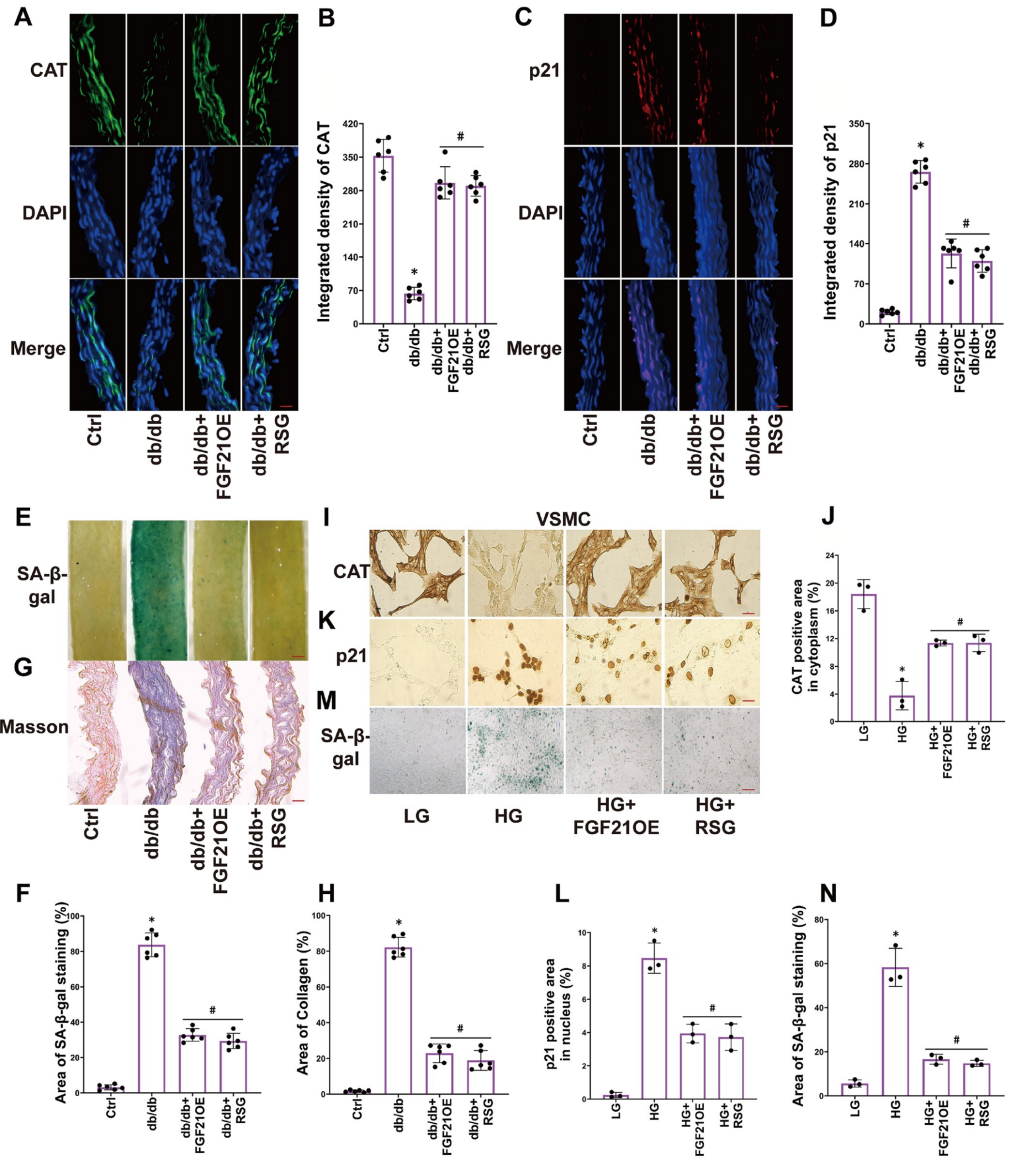
Figure 3 FGF21 activates the PPARγ-CAT pathway to alleviate senescence in the vascular smooth muscle layer of diabetic mice and HG-treated VSMCs (A,B) Representative immunofluorescence images of CAT (400×, scale bar: 20 μm) and the summarized data of CAT integrated density in aortas. (C,D) Representative immunofluorescence images of p21 (400×, scale bar: 20 μm) and the summarized data of p21 integrated density in aortas. (E‒H) Representative images of SA-β-gal staining (40×, scale bar: 200 μm) and Masson staining (400×, scale bar: 20 μm), the summarized data of SA-β-gal (green) and collagen deposition (blue) area percentage. (I‒L) Representative IHC images of CAT, p21 (400×, scale bar: 20 μm) and the summarized data of CAT, p21 positive (yellow) area in VSMCs. (M,N) Representative images of SA-β-gal staining (200×, scale bar: 40 μm) and summarized data of SA-β-gal (green) area percentage in VSMCs. n=6 in mice; n=3 in VSMCs. *P<0.05 vs Ctrl; #P<0.05 vs db/db or HG group.

To investigate the pivotal role of PPARγ in diabetes-induced senescence of the vascular smooth muscle layer, we activated the PPARγ pathway in diabetic mice and vascular smooth muscle cells (VSMCs) through treatment with RSG (a known PPARγ agonist). We observed that RSG restored the expression of CAT ( Figure 3A,B), suppressed the expression of p21 ( Figure 3C,D) in the blood vessel wall, and reduced the area of SA-β-gal (green staining) ( Figure 3E,F) and collagen deposition (blue staining) ( Figure 3G,H). Similar results were obtained in VSMCs, where RSG restored the HG-induced impairment of CAT expression ( Figure 3I,J), suppressed the HG-induced increase in p21 expression ( Figure 3K,L), and inhibited the green-stained area of SA-β-gal ( Figure 3M,N). FGF21 overexpression had effects similar to those of RSG ( Figure 3A–N).These results indicate that FGF21 alleviates senescence in the vascular smooth muscle layer of diabetic mice and HG-treated VSMCs by activating the PPARγ-CAT pathway.

### FGF21 protects PPARγ and alleviates senescence in the vascular smooth muscle layer of diabetic mice and HG-treated VSMCs by suppressing NLRP3

**Figure 4:**
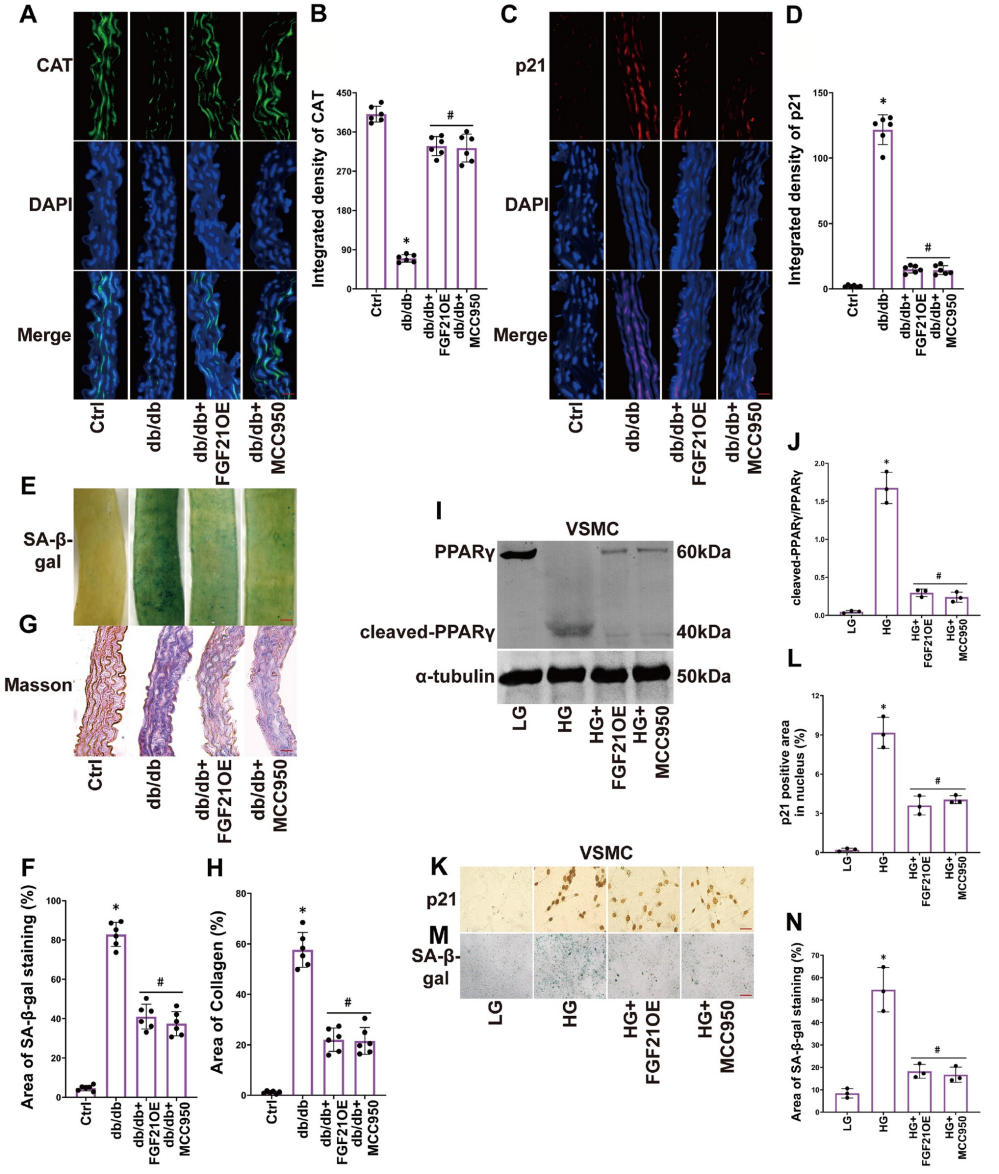
Figure 4 FGF21 protects PPARγ and alleviates senescence in the vascular smooth muscle layer of diabetic mice and HG-treated VSMCs by suppressing NLRP3 (A,B) Representative immunofluorescence images of CAT (400×, scale bar: 20 μm) and the summarized data of CAT integrated density in aortas. (C,D) Representative immunofluorescence images of p21 (400×, scale bar: 20 μm) and the summarized data of p21 integrated density in aortas. (E‒H) Representative images of SA-β-gal staining (40×, scale bar: 200 μm) and Masson staining (400×, scale bar: 20 μm), the summarized data of SA-β-gal (green) and collagen deposition (blue) area percentage in aortas. (I,J) Representative western blot images and the summarized data of the cleavage of PPARγ in VSMCs. (K‒N) Representative IHC images of p21 (400×, scale bar: 20 μm) and images of SA-β-gal staining (200×, scale bar: 40 μm) and summarized data of p21-positive (yellow) area and SA-β-gal (green) staining area percentage in VSMCs. n=6 in mice; n=3 in VSMCs. *P<0.05 vs the Ctrl; #P<0.05 vs the db/db- or HG-treated group.

To explore the critical role of NLRP3 in diabetes-induced senescence of the vascular smooth muscle layer, we inhibited NLRP3 in diabetic mice and VSMCs through MCC950 treatment. We observed that MCC950 restored the expression of CAT ( Figure 4A,B), suppressed the expression of p21 ( Figure 4C,D) in the blood vessel wall, reduced the green-stained area of SA-β-gal ( Figure 4E,F), and decreased the deposition of blue-stained collagen ( Figure 4G,H). Similar results were obtained in VSMCs, where MCC950 alleviated the PPARγ cleavage triggered by HG ( Figure 4I,J), suppressed the expression of p21 induced by HG ( Figure 4K,L), and inhibited the green-stained area of SA-β-gal ( Figure 4M,N). FGF21 overexpression produced results comparable to those of MCC950 ( Figure 4A–N).These results demonstrate that FGF21 mitigates senescence in the vascular smooth muscle layer of diabetic mice and HG-treated VSMCs by inhibiting the NLRP3 pathway.

### FGF21 protects PPARγ and CAT, alleviating senescence in the vascular smooth muscle layer of diabetic mice and HG-treated VSMCs by inhibiting the SYK pathway

**Figure 5:**
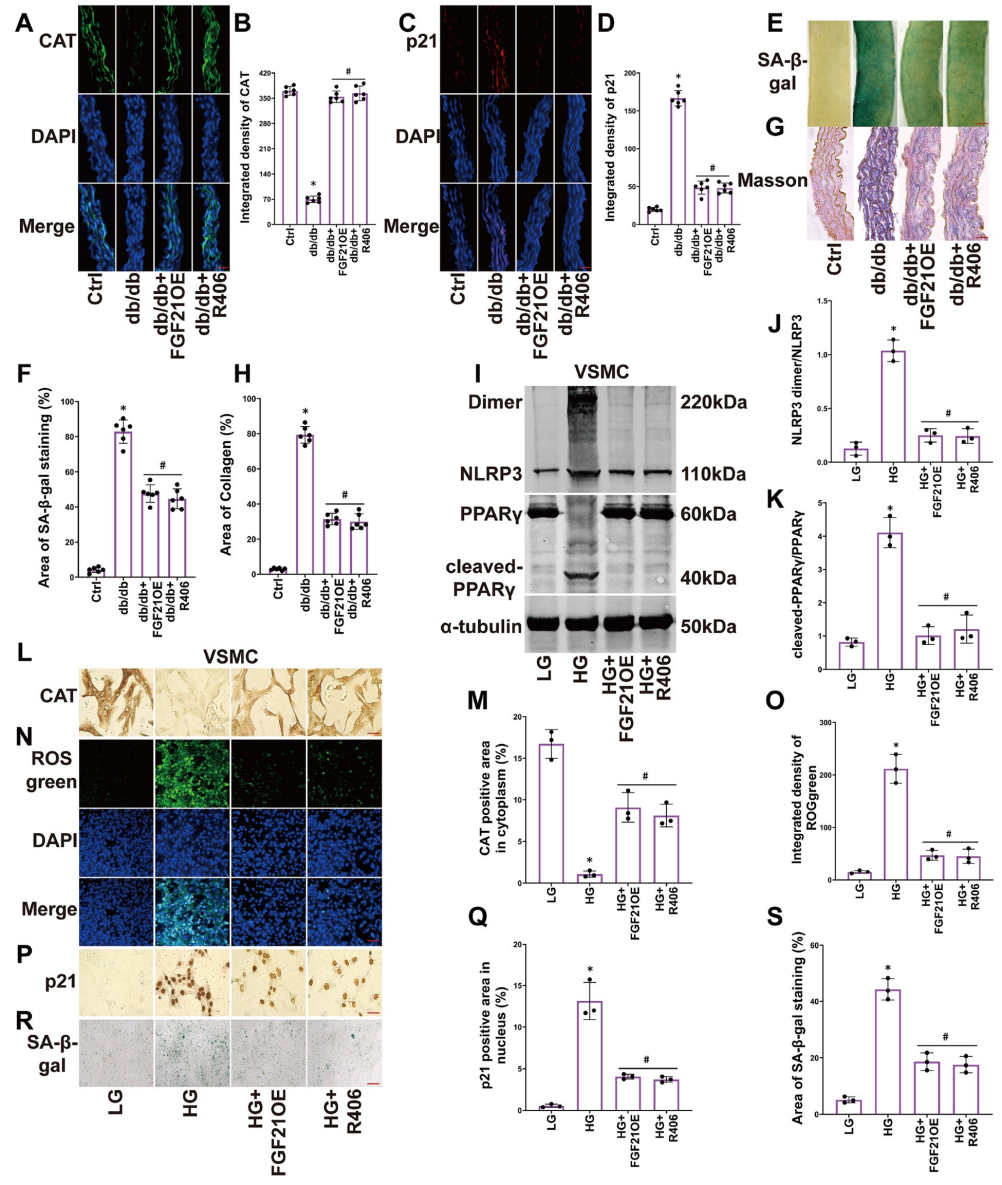
Figure 5 FGF21 protects PPARγ and CAT, alleviating senescence in the vascular smooth muscle layer of diabetic mice and HG-treated VSMCs by inhibiting the SYK pathway (A,B) Representative immunofluorescence images of CAT (400×, scale bar: 20 μm) and the summarized data of CAT integrated density in aortas. (C,D) Representative immunofluorescence images of p21 (400×, scale bar: 20 μm) and the summarized data of p21 integrated density in aortas. (E‒H) Representative images of SA-β-gal staining (40×, scale bar: 200 μm) and Masson staining (400×, scale bar: 20 μm), the summarized data of SA-β-gal (green) and collagen deposition (blue) area percentage in aortas. (I‒K) Representative western blot images and the summarized data of the production of the NLRP3 dimer and the cleavage of PPARγ in VSMCs. (L,M) Representative IHC images of CAT (400×, scale bar: 20 μm) and the summarized data of the CAT-positive (yellow) area in VSMCs. (N,O) Representative images of ROSgreen staining (200×, scale bar: 40 μm) and the summarized data of ROSgreen integrated density. (P,Q) Representative IHC images of p21 (400×, scale bar: 20 μm) and the summarized data of the p21-positive (yellow) area in VSMCs. n=6 in mice; n=3 in cells. *P<0.05 vs Ctrl; #P<0.05 vs db/db or HG-treated group.

To explore the underlying role of SYK in diabetes-induced senescence of the vascular smooth muscle layer, we utilized the SYK-specific inhibitor R406 in diabetic mice and VSMCs to block the SYK pathway. We found that R406 restored the expression of CAT ( Figure 5A,B), suppressed the expression of p21 in the blood vessel wall ( Figure 5C,D), limited the green-stained area of SA-β-gal ( Figure 5E,F), and reduced blue-stained collagen deposition ( Figure 5G,H). Furthermore, in VSMCs, R406 reversed the HG-induced NLRP3 dimerization ( Figure 5I,J) and PPARγ cleavage ( Figure 5I,K), restored HG-impaired CAT expression ( Figure 5L,M), inhibited the integrated ROSgreen fluorescence intensity ( Figure 5N,O), suppressed HG-induced p21 expression ( Figure 5P,Q), and decreased the green-stained area of SA-β-gal ( Figure 5R,S). FGF21 overexpression mimicked the effects of R406 treatment ( Figure 5A–S).These results indicate that FGF21 alleviates senescence in HG-induced VSMCs by inhibiting the SYK-NLRP3 pathway to protect PPARγ and CAT.

## Discussion

This study revealed that FGF21 protects the PPARγ-CAT pathway by inhibiting the SYK-NLRP3 inflammasome pathway, thereby alleviating the accumulation of hydrogen peroxide in VSMCs under HG conditions and attenuating the senescence of the vascular smooth muscle layer in diabetic mice. This study suggested that the generation of the NLRP3 dimer may play a crucial role in the assembly and activation of the NLRP3 inflammasome. Additionally, this study is the first to observe the alleviation of diabetes-induced VSMC senescence by FGF21 and to investigate its potential mechanisms. The findings of this study provide novel insights into the mechanisms underlying premature vascular senescence induced by the diabetic pathological environment and provide additional evidence for the vascular protective effects of FGF21.

Some studies have demonstrated that FGF21 mitigates vascular wall calcification and remodeling by counteracting oxidative stress [ 29, 30]. However, the mechanisms responsible for the antioxidant effects of FGF21 remain incompletely understood. Our study revealed that FGF21 maintains CAT level by protecting PPARγ, thereby reducing the accumulation of hydrogen peroxide. This conclusion provides new evidence for the role of FGF21 in counteracting the increase in oxidative stress induced by diabetes. FGF21 is believed to play a role in alleviating the senescence of mesenchymal stem cells, chondrocytes, and neural cells, and its mechanisms are often associated with the inhibition of ROS levels [ 31– 33]. Some studies have suggested that FGF21 contributes to alleviating vascular senescence. FGF21 delays the occurrence of hydrogen peroxide-induced EC senescence by protecting the SIRT1 pathway [34]; FGF21 inhibits the AMPK-p53 pathway to suppress the ROS induced by angiotensin II, thereby alleviating the senescence of cerebral vascular smooth muscle cells [20]. However, the protective effects of FGF21 on the senescence of the peripheral vascular smooth muscle layer, particularly in the context of diabetic pathological conditions, still require further research and elucidation. Our findings contribute to filling this research gap, as we demonstrate that FGF21 overexpression effectively alleviates the senescence of the aortic vascular smooth muscle layer induced by a diabetic pathological environment characterized primarily by high blood glucose levels. This protective effect is accompanied by the inhibition of excessive collagen deposition and the restoration of impaired vascular relaxation. The findings of the present study revealed the novel pharmacological effects of FGF21 in protecting VSMCs, specifically by alleviating the senescence of the smooth muscle layer induced by diabetes.

It is generally recognized that the upregulation of ROS levels is one of the main mechanisms of NLRP3 inflammasome activation. Some studies have indicated that ROS-induced NLRP3 inflammasome activation induces EC dysfunction and VSMC calcification [ 15, 35]. Our study showed that HG-induced NLRP3 inflammasome activation leads to decreased CAT expression in VSMCs, the accumulation of hydrogen peroxide, and the upregulation of ROS. These results suggest that NLRP3 inflammasome activation induces the upregulation of ROS levels, which differs from the aforementioned findings. These fragmented results may support our conclusion. For example, in the treatment of multiple sclerosis, the restoration of CAT level was observed while inhibiting NLRP3 inflammasome activation [36]; in the study of airway epithelial cell injury, the restoration of CAT expression was observed while inhibiting NLRP3 inflammasome-associated RNA and protein expression [37]. Additionally, studies have shown that knockout of *NLRP3* reduces ROS production, protecting human aortic endothelial cells [38]. Our research findings, together with these existing research results, may suggest the occurrence of ROS-NLRP3 inflammasome-CAT-ROS cycle. We believe that this vicious cycle is likely to be one of the key mechanisms of cell damage and senescence mediated by ROS and the NLRP3 inflammasome, which is worth exploring further.

Furthermore, it has been reported that the production of NLRP3 oligomers is a necessary initial step in NLRP3 inflammasome assembly [ 39, 40]. Surprisingly, in this study we observed the occurrence of NLRP3 dimerization. We highly suspect that the formation of the NLRP3 dimer may play a significant role in the activation of the NLRP3 inflammasome induced by HG in VSMCs. Our study provides new insight into a novel mechanism by which HG induces NLRP3 inflammasome activation.

The inhibitory effect of FGF21 on the NLRP3 inflammasome has been confirmed. FGF21 protects the vascular endothelium and smooth muscle layers by inhibiting the NLRP3 inflammasome [ 41, 42], and our previous research showed that FGF21 inhibits VSMC proliferation and migration through inhibition of the SYK-NLRP3 inflammasome pathway, alleviating diabetes-induced neointimal hyperplasia [19]. The present research findings reinforce the possibility that SYK serves as a pivotal pathway through which FGF21 mitigates NLRP3 inflammasome activation. In the current study, we further observed that FGF21 inhibits SYK phosphorylation-mediated NLRP3 dimerization, thereby suppressing NLRP3 inflammasome activation to protect the PPARγ-CAT pathway. This protective mechanism limits oxidative stress and mitigates HG-induced VSMC senescence. This finding provides a novel perspective on the inhibition of SYK-induced ROS production and the NLRP3 inflammasome by FGF21.

Studies have indicated the diverse roles of the PPARγ pathway in cellular senescence [ 43, 44]. Our findings support the view that the PPARγ pathway has a protective effect on mitigating VSMC senescence. This finding is somewhat similar to the conclusions of other studies showing that PPARγ agonists alleviate cellular senescence [ 45, 46]. However, the role of the PPARγ pathway in VSMC senescence still requires further investigation. PPARγ is cleaved by the core product of the NLRP3 inflammasome, caspase-1, and this cleavage plays a critical role in inducing TAM infiltration [ 47, 48]. We hypothesize that this cleavage effect may also play a role in diabetic vascular lesions. Our results indicate that PPARγ cleavage coincides with the downregulation of CAT level and the senescence of the vascular smooth muscle layer and that activation of the PPARγ pathway slows HG-induced VSMC senescence. We propose that blockade of the NLRP3 inflammasome in the PPARγ-CAT pathway may induce VSMC senescence under hyperglycemic conditions. We speculate that NLRP3 inflammasome-mediated PPARγ cleavage could be a novel target for diabetic vascular lesions and warrants further investigation.

FGF21 upregulates the PPARγ pathway to alleviate inflammation in microglia and macrophages and protect brain microvascular endothelial cells, playing a positive role in stroke [ 49, 50]. FGF21 activation of the PPARγ pathway eliminates the production of inflammatory factors, suppresses pulmonary artery smooth muscle cell proliferation, and alleviates pulmonary arterial hypertension [ 51, 52]. These findings imply that the FGF21-PPARγ pathway has a protective effect on VSMCs. Our findings support this conclusion, as our results show that FGF21 increases PPARγ level and alleviates VSMC senescence. However, further studies are needed to investigate the effect of the FGF21-PPARγ pathway on other VSMC processes, such as proliferation, migration, apoptosis, and calcification.

In conclusion, our study revealed a novel mechanism by which HG conditions induce NLRP3 protein dimerization, triggering NLRP3 inflammasome assembly and activation, leading to downregulation of PPARγ-mediated CAT expression, accumulation of hydrogen peroxide, and accelerated VSMC senescence. Overexpression of FGF21, through the inhibition of HG-induced SYK phosphorylation and NLRP3-mediated PPARγ cleavage, reduces CAT level and alleviates oxidative stress level, reducing vascular smooth muscle layer senescence in diabetic mice. Our study reveals a new mechanism by which diabetes accelerates vascular senescence and identifies the pharmacological effect of FGF21 in mitigating diabetes-induced vascular senescence, providing new evidence for its potential clinical application.

## Supporting information

513FigS1
