# Supplementary material for: FGF21 overexpression alleviates VSMC senescence in diabetic mice by modulating the SYK-NLRP3 inflammasome-PPARγ-catalase pathway: FGF21 alleviates high glucose-induced VSMC senescence
Source: Acta Biochim Biophys Sin (Shanghai). 2024 May 10;56(6):892–904. doi: 10.3724/abbs.2024032 (PMC11214975; doi:10.3724/abbs.2024032)
Supplement: 513FigS1 [file 513FigS1.pdf]

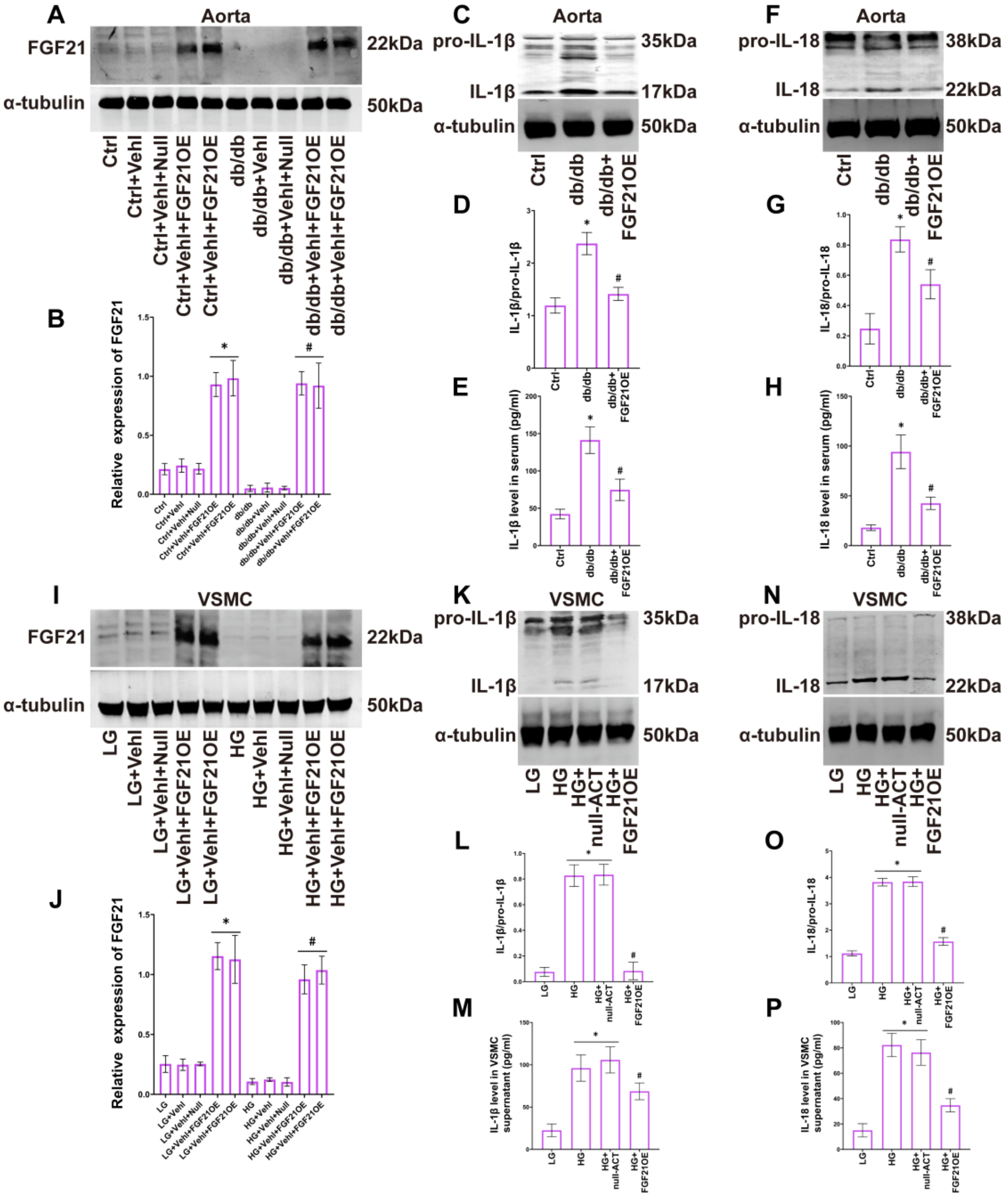


**Supplementary Figure S1. FGF21 expression is activated by transfection in mice and VSMCs, and FGF21OE inhibits the production and secretion of active IL-1β and IL-18 in diabetic mice and HG-induced VSMCs** (A,B) Representative western blot images and the summarized data of FGF21 in nondiabetic or diabetic aortas. (C–E) Representative western blot images and the summarized data of IL-1β/pro-IL-1β in aortic homogenate and the IL-1β level in serum detected by ELISA. (F–H) Representative western blot images and the summarized data of IL-18/pro-IL-18 in aortic homogenate and the IL-18 level in serum detected by ELISA. (I,J) Representative western blot images and the summarized data of FGF21 in regular or HG-treated VSMCs. (K–M) Representative western blot images and the summarized data of IL-1β/pro-IL-1β in the VSMC lysate and the IL-1β level in the VSMC supernatant detected by ELISA. (F–H) Representative western blot images

and the summarized data of IL-18/pro-IL-18 in the VSMC lysate and the IL-18 level in the VSMC supernatant detected by ELISA. $n$=3. *$P$<0.05 vs the control Ctrl; $^{\#}P$<0.05 vs the db/db or HG treated group.
